# Development of nutrient-rich complementary foods using locally sourced ingredients for low-income households in Eastern Ethiopia

**DOI:** 10.3389/fnut.2025.1537357

**Published:** 2025-03-05

**Authors:** Anbesse Girma Shewa, Shewangizawe Teketele Anamoo, Solomon Abera, Mikiyas Kebede Ali, Jalene Gelan

**Affiliations:** ^1^Department of Food Science and Postharvest Technology, Haramaya Institute of Technology, Haramaya University, Harar, Ethiopia; ^2^Department of Food Technology and Process Engineering, Haramaya Institute of Technology, Haramaya University, Harar, Ethiopia

**Keywords:** *β*-carotene, infant formula, spinach leaf powder, malnutrition, orange-fleshed sweet potato

## Abstract

**Background:**

The prevalence of malnutrition among infants continues to be a significant issue in Ethiopia. Although commercial complementary foods are accessible in the market, their prohibitive costs render them unaffordable for low-income households. Consequently, this study was undertaken to formulate complementary foods utilizing locally available, nutrient-rich ingredients tailored for low-income households.

**Method:**

The effect of various processing techniques—such as boiling, germination, and roasting—on the physicochemical properties of maize and groundnut flours, including proximate composition, mineral content, phytochemicals, and β-carotene levels, was evaluated using standard methodologies. A complementary food product was developed by varying the blending ratios of maize flour, groundnuts, spinach leaves, and sweet potatoes. Subsequently, the physicochemical and sensory characteristics of the product were assessed.

**Results:**

The processes of germination and roasting (applied to maize and groundnut) demonstrated minimal impact on the proximate composition, mineral content, and β-carotene levels while also decreasing the amounts of specific phytochemicals (such as tannins, phenols, and phytic acid) found in the raw materials. Consequently, these processed ingredients were used to formulate eight complementary food products. The results of the proximate composition analysis for the eight developed food formulas indicated that the protein content ranged from 15.35 to 16.39%. Additionally, the fat, carbohydrate, and energy values were observed to range from 8.1 to 11.9%, 59.12 to 63.07%, and 383.82 to 412.87 kcal, respectively, indicating a nutritional profile consistent with locally available commercial complementary foods. Similarly, the levels of calcium, zinc, iron, magnesium, and β-carotene were measured to range from 66.75 mg to 102.48 mg, 1.33 mg to 2.48 mg, 6.64 mg to 10.36 mg, 122.60 mg to 181.73 mg, and 113.40 mg to 197.53 mg per 100 g, respectively, alongside notably low levels of anti-nutritional factors.

**Conclusion:**

Including supplementary food is crucial when breastfeeding alone does not adequately meet an infant’s nutritional requirements. As a result, the complementary food developed can provide 5 to 50% of the daily recommended nutrient allowance for infants.

## 1 Introduction

The prevalence of food and nutrition insecurity among women, children, and infants continues to be a significant issue in many sub-Saharan countries, including Ethiopia (1, 2). Malnutrition among women, children, and infants poses a critical challenge within the broader community in Ethiopia (3). The World Health Organization (WHO) report indicates that, despite some observed progress, approximately 21.6% of the population in Ethiopia is undernourished, with infants and children being the most vulnerable groups (4). Over 14% of pregnant women and infants suffer from maternal and infant malnutrition, which has contributed to a significant number of maternal and infant deaths in the country (3, 5). This issue is exacerbated among infants and children, particularly infants, as they often do not receive a variety of nutrient-rich complementary foods to fulfill their daily nutritional needs.

After 6 months of age, infants become ready to consume pureed, mashed, and semi-solid foods made from cereals, vegetables, fruits, meats, and various protein-rich sources (6, 7). As oral skills develop (such as chewing and munching), the texture of complementary foods can gradually shift to include ground and diced options (8). Lumpy solid foods should be introduced around 10 months of age to minimize potential feeding risks (9).

The nutrient compositions of most food products are inadequate to meet the nutritional requirements of all age groups, including infants (10). In Eastern Hararghe, daily diets across all age groups rely heavily on certain products, with sorghum budena being the most common staple dish consumed daily (11). Foods designed for infants face specific challenges regarding nutritional value, as they are limited and contain low levels of vitamins and health-promoting compounds. Consequently, infants who depend on such diets are experiencing malnutrition and related health issues. Deficiencies in vitamins and minerals remain a prevalent public health concern in Eastern Hararghe (12).

Although high-quality commercial products are sometimes available, they are often prohibitively expensive for low-income rural households. Alternative strategies are crucial to enable families to nourish their infants with better formulations using locally available staples. Therefore, it is vital to create complementary food options from locally sourced, appropriate, and affordable food items that can reduce malnutrition and improve the health of infants and children. These items may play a key role in effective and locally tailored nutritional interventions aimed at addressing malnutrition (13). Locally sourced, nutrient-rich foods have the potential to enhance the nutritional status of infants, as they provide readily available and affordable sources of essential nutrients for low-income households (14).

For the current development of complementary food, raw materials such as maize, a source of carbohydrates (15), bio-fortified orange-fleshed sweet potato (OFSP), a source of β-carotene (16), groundnut, a source of protein and fat (15), and spinach leaves, a source of minerals (17), were selected.

Thus, the research aimed to create complementary foods from locally available sources, including cereals, legumes, roots, and leafy vegetables, to improve energy, protein quality, and mineral and vitamin content.

## 2 Materials and methods

### 2.1 Study materials

The raw materials (maize grain, orange-fleshed sweet potato, and groundnut) were sourced from the Haramaya University research center and Babile model farmers. Fresh spinach leaves were acquired from the local market in Bate town. All raw materials were cultivated during the country’s rainy season (June to September) and harvested in November 2024 based on quality characteristics, including uniformity in size, absence of damage, and freedom from disease. The harvesting periods for maize, orange-fleshed sweet potato, groundnut, and spinach leaves were 4.5, 3.5, 3, and 1.5 months, respectively. After collection, the samples were transported to the laboratory and stored appropriately until further processing.

### 2.2 Sample preparation

#### 2.2.1 Orange-fleshed sweet potato flour

The flour from orange-fleshed sweet potatoes (*Ipomoea batatas [L.] Lam*) was prepared using Truong et al.’s (18) method with minor modifications. During preparation, two kilograms of tubers, free of dirt and contaminants, were thoroughly cleaned with potable water and manually peeled with a kitchen knife. The peeled tubers were then sliced into smaller pieces using a hand-operated slicer, achieving a uniform thickness of 2 mm. The slices were rinsed and placed in a stainless-steel pot, then blanched in distilled water on a hot plate at 85°C for 5 min. After blanching, the tuber slices were drained, rinsed, spread out on trays, and dried in a hot oven dryer at 60°C for 12 h with occasional stirring to ensure even drying. The dried slices were milled with an electric powder grinder (DE 100 g) and passed through a 500-μm mesh sieve. Finally, the flour was packed in an airtight high-density polyethylene bag, labeled, and stored in a dry place until further use and analysis.

#### 2.2.2 Maize flour

Maize (*Zea mays L.*) was cleaned, sorted, and grouped into three batches, each containing 3 kg of maize. Then, each batch underwent roasting, malting, and boiling treatment.

Roasted maize flour was prepared using the method described by Shiriki et al. (15). The clean maize was washed separately in fresh tap water and air-dried for 12 h. The maize was then roasted in an oven at 70°C for 30 min, after which the seed coat was removed through dehulling. The maize was milled with an electric grinder (DE 100 g) and sieved through a 500-μm sieve. The resulting flour was packaged in a flexible, airtight, high-density polyethylene bag, labeled, and stored in a dry place until needed for further use.

For maize malting, the maize was pretreated for 5 min with 1.25% sodium hypochlorite mixed in distilled water to control microbial growth. It was then rinsed and soaked in distilled water (1:3, w/v) for 9 h at room temperature. After draining, the maize was placed on perforated aluminum pans lined with cloth bags and positioned in a dark, temperature-controlled cabinet (model KBP termaks Norway) at 30°C and 85% relative humidity for germination. After 4 days of germination, the maize was manually washed with distilled water, oven-dried at 60°C for 20 h, and finally milled using an electric grinder (DE 100 g). The flour was then packed in an airtight, high-density polyethylene bag and stored at room temperature.

The third batch of maize was cleaned, boiled in water, drained, mashed into a paste, and dried at 50°C for 24 h. The dried sample was then ground and sieved using a 500-μm sieve, packed in an airtight high-density polyethylene bag, and stored in a dry place at room temperature until further laboratory work.

#### 2.2.3 Germinated groundnut flour

The preparation of germinated groundnut was conducted in accordance with the methodology outlined by Anandan et al. (19), with minor modifications. The groundnut (*Arachis hypogaea*) was meticulously cleaned, sorted, and categorized into three distinct batches, each weighing 3 kg.

The first batch was manually cleaned to remove foreign materials and soaked in a 70% ethanol solution for 15 min at room temperature for disinfection. After rinsing the seeds with tap water and distilled water to remove the ethanol, the seeds were soaked in distilled water (1:5, w/v) for 12 h at room temperature. The water was drained, and the seeds were placed between thick layers of cotton cloth, allowing them to germinate for about 1 week in a temperature-controlled cabinet (model KBP termaks Norway) at 30°C with 85% relative humidity. The seeds were moistened daily with fresh distilled water. The germinated groundnuts were then dried in an oven at 50°C for 24 h. After the dehulling process, the dried beans were ground to pass through a 500-μm mesh sieve and stored in an airtight high-density polyethylene plastic bag in a dry place until further analysis.

The second batch was cleaned and roasted in a conventional oven using the method developed by Ferreira et al. (20). Raw groundnut seeds were roasted at 160°C for 12 min. After roasting, the kernels were cooled to room temperature, then de-hulled and milled. Finally, the third batch was boiled in clean water at a ratio of 1:10 (w/v). The boiled sample was drained and mashed into a paste, then dried at 50°C for 24 h. Similarly, the dried sample was ground through a 500-μm mesh sieve and stored in an airtight high-density polyethylene plastic bag until further laboratory work.

#### 2.2.4 Spinach flour

The preparation of spinach flour was conducted in accordance with the methodology established by Galla et al. (21), with minor modifications. A total of 5 kg of fresh spinach leaves (*Spinacia oleracea*) underwent preliminary grading, followed by washing to eliminate any dirt. Subsequently, the leaves were immersed in a 40-ppm sodium hypochlorite solution for 30 min to ensure disinfection. Any residual sodium hypochlorite was eliminated by rinsing the leaves with potable water. The leaves were then shredded and dried in an oven dryer (model PF120 (200) Carbolite England) at a temperature of 45°C for 12 h. The dried leaves were ground using an electric grinder (DE 100 g) and sieved through a 500-μm sieve. The resulting powder was then packed into airtight polyethylene bags and stored in a dry environment until required for experimentation.

### 2.3 Development of complementary food

The formulation was derived from literature that addresses the nutritional requirements of complementary foods for infants. A ratio was assigned to each raw material to enhance the nutritional composition of the final product while minimizing any adverse effects on product quality (22–25). The flour blends were produced from the formulations (F), following the proportions indicated in Table 1. The formulations from F1 to F4 were made with roasted maize and groundnut, whereas F5 to F8 utilized germinated maize and groundnut. The choice of processing methods (roasting and germination) was based on their effectiveness in demonstrating the superior chemical, physical, and functional properties of the flour.

**Table 1:** Total blending combinations in eight complementary foods developed.

Formula (F)	Blending ratio (%)			
	Roasted maize	Roasted groundnut	OFSP	Spinach
F1	60	15	10	10
F2	55	10	15	15
F3	50	15	20	10
F4	45	10	25	15
	Germinated maize	Germinated groundnut	OFSP	Spinach
F5	60	15	10	10
F6	55	10	15	15
F7	50	15	20	10
F8	45	10	25	15

Each formulation yielded half a kg of a blend, which was then dried in the oven at 60°C for 2 h at 60°C after incorporating 4.5% sugar and 0.5% salt. The final products were packaged in high-density polyethylene bags and stored in a clean, dry, and cool place.

### 2.4 Analysis of proximate compositions

#### 2.4.1 Proximate composition

The standard method of the American Association of Cereal Chemists (26) was used for the determination of moisture content, crude protein, crude fat, ash content, and crude fiber content of flour. In total, 3 g of sample was used for all proximate composition, except for protein, which was 5 g, and analysis was conducted in triplicate.

##### 2.4.1.1 Moisture content

After preparing a clean and dry dish, its weight was taken as W_1_. A sample of flour of 3 g was weighed (W_2_) after being placed on the dish, then dried at 100°C for 6 h until constant weight was obtained, and cooled to room temperature in desiccators. The mass after drying was then measured as W_3._ Finally, the moisture content of flour was calculated by using the following formula:


$$M.C=\left(\frac{W_{2}-W_{3}}{W_{2}-W_{1}}\right)\times 100,$$

where M.C is moisture content (%), W_1_ is the mass of the dish (g), W_2_ is the mass of the sample and dish before drying (g), and W_3_ is the mass of the sample and dish after drying (g).

##### 2.4.1.2 Crude protein

The sample (5 g) was added to a Kjeldahl digestion flask with a catalyst mixture (H2SO4 mixed with selenium in a 100-mL volumetric flask) with 7.2 g of salicylic acid solutions formed. After adding 2.5 mL of catalyst mixture solution into each sample (digestion flask), it was placed in the digester (Model DK-20F30100184, Velp Scientific, Italy), and the temperature was brought to 100°C. It was allowed to digest for over 2 h until completed. The digesters’ flask was removed and let to cool. After it was cooled, the contents of the flask were diluted with 30 mL of distilled water followed by 25 mL, and 40% NaOH was added to the digestion flask, neutralizing the acid and mildly alkalizing the solution. The contents were distilled (Model UDK 129, VELP Scientifica, Italy) immediately by putting the digestive tube into the receiver flask containing 25 mL of 4% boric acid solution, yielding approximately 150 mL of distillate. Finally, a standard acid (ca. 0.1 N HCl) was used to titrate the distillate. Using the proper factor, the percent of nitrogen (N) was transformed to percent protein (protein = 6.25 × %N). In the analysis, urea was used as a control.


$$N=\frac{V_{HCl}\times N_{HCl}\times 14}{m}\times 100$$

P=F×N, Where V _HCl_ is the volume of HCl consumed during the endpoint of the titration, N _HCl_ indicates the normality of HCl (commonly 0.1 N), m stands for the sample weight on a dry matter basis, 14.00 is the molecular weight of nitrogen, N is nitrogen (%), F is the conversion factor (6.25), and P is protein (%).

#### 2.4.2 Utilizable carbohydrate

The carbohydrate content of the samples was determined by subtracting the total of other constituents from 100.

% carbohydrate = 100 – (% moisture content + % crude protein + % fiber + % crude fat + % ash).

#### 2.4.3 Energy

The energy density (DE) of the samples was calculated based on the proximate composition using a factor of 4 kcal/g for both carbohydrates and proteins and 9 kcal/g for lipids.

DE = [9 x lipids (%) + 4 x carbohydrates (%) + 4 x proteins (%)].

### 2.5 Determination of mineral contents

Atomic absorption spectrometry (Model 230ATS AA, Buck Scientific, USA) was used to determine the calcium, iron, zinc, and phosphorus content of raw materials and the product samples (26). The mineral content of samples was reported on a dry weight basis.

### 2.6 Determination of anti-nutritional and beta carotene content

#### 2.6.1 Tannin

The modified vanillin-HCl methanol technique, utilizing Price et al.’s (27) vanillin-HCl method, was used to *determine condensed* tannin.

The tannin content was calculated as catechin equivalents:


$$\text{Tannin}\left(\%\right)=\frac{\left(C\times 10\right)\times 100}{200}$$

#### 2.6.2 Phytic acid

A sample of 2 g was placed in a 250-mL conical flask and soaked for 3 h in 100 mL of 2% concentrated hydrochloric acid. The mixture was then filtered twice through hardened filter paper. To achieve the desired acidity, 50 mL of each filtrate was transferred to a 250-mL beaker, and 107 mL of distilled water was added. As an indicator, 10 mL of 0.3% ammonium thiocyanate solution was added to each solution. The mixture was then titrated with a 0.00195 g iron per mL standard iron (iii) chloride solution. The endpoint was slightly brownish-yellow and lasted 5 min (27). The percentage of phytic acid was calculated using the following formula:


$$\%\text{phytic acid}=\frac{\left(X.1.19.100\right)}{2},$$

Where *X* = Titer value x 0.00195.

#### 2.6.3 Total phenol

Total phenolic content was determined according to Tupe et al. (28). A sample of 200 mg was extracted with 4 mL of acidified methanol (HCL/methanol/water, 1:80:10 V/V/V) at room temperature (25°C) for 2 h. An aliquot of the extract (200 μL) was added to 1.5 mL of freshly diluted (10-fold) Folin–Ciocalteu reagent. The mixture was allowed to equilibrate for 5 min and then mixed with 1.5 mL of sodium carbonate solution (60 g/L). After incubation at room temperature (25°C) for 90 min, the absorbance of the mixture was measured at 725 nm using a UV/Vis spectrophotometer (Model: AAS 700, Perkin Elmer, USA). Acidified methanol served as the blank sample. A series of six standard solutions (0, 5, 10, 15, 20, 25, 30, and 35 ppm) was prepared from the stock solution of standard gallic acid. The total phenolic was estimated from the calibrated curve as gallic acid equivalent (GAE) per kilogram of sample. The phenolic content was then calculated using the following formula:


$$\text{Total phenolic}\;\left(\text{ppm}\right)=\frac{\left(\mathrm{\mu g}/\text{mL}\right)\text{xDfx}100}{\text{Sample mass}\;\left(db\right)},$$

Where μg/mL is the absorbance reading concentration, and Df is the dilution factor.

#### 2.6.4 Determination of beta carotene content

It was determined using the standard procedure outlined in AACC International (26) Method 14–50 with a spectrophotometer (Model: AAS 700, Perkin Elmer, USA). A 3 g sample, milled to a fineness of 0.5 mm, was analyzed after pigment extraction using 15 mL of water-saturated n-butyl alcohol (1:5, v/v). The absorbance of the extract was read at 435.8 nm against water-saturated butanol as a blank. A 1 mg pigment sample was dissolved in 100 mL, measuring the water-saturated n-butanol, which has an absorbance (optical density) of 1.66632 in a 1.0-cm cuvette. Consequently, the pigment mass in the sample was calculated using the equation.


$$\text{Sample pigment mass}\;\left(\text{mg}/g\right)=\frac{\text{sample}\;\text{Abs}x0.15}{1.6632}$$

### 2.7 Determination of functional properties

The water absorption index, bulk density, and solubility index were analyzed according to Eriksson’s (29) method, and the oil absorption index was based on Julianti et al.’s (30) method.

### 2.8 Sensory acceptability

The Nine-Point Hedonic Scale taste method of Alshehry (31) was followed to conduct the sensory evaluation of complementary food after rehydrating it with boiled water. The rating was scaled as 1 = dislike extremely, 2 = strongly disliked, 3 = moderately disliked, 4 = slightly disliked, 5 = neither like nor dislike, 6 = slightly liked, 7 = moderately liked, 8 = strongly liked, and 9 = like extremely. It was performed using thirty (30) semi-trained panelists drawn from local women and students (undergraduate, postgraduate, and instructors) from the Food Science and Technology Department, Haramaya University.

The purpose of the test was explained to the panelists, and their health status was taken into consideration during selection to ensure they did not have colds and allergies that could affect their sensitivity to the product. The formula (~75 g) was rehydrated with 220 mL boiled water and stirred until a smooth, thin gruel was obtained, allowed to cool slightly to a suitable tasting temperature. Samples were coded with three-digit numbers and spaced apart to prevent crowding, ensuring independent judgment. The gruel was then served to semi-trained women. The panelists were asked to test the gruel using the Nine-Point Hedonic Scale. Between each test, the panelists were instructed to rinse their mouths with tap water before proceeding to the next sample.

### 2.9 Data analysis

Data arrangement, organization, and basic descriptive statistical analyses were conducted using an Excel spreadsheet. All experimental analyses and product development formulas were carried out in triplicate. The data were exported to statistical software for further analysis. All data were analyzed for variance (one-way ANOVA) among treatments using R-studio software (version 4.3.3). Mean separation was conducted using Duncan’s multiple range tests, with significance determined at a *p*-value of <0.05. Tables and graphs were employed to present the findings of the current study.

## 3 Results

### 3.1 Chemical compositions of raw materials

#### 3.1.1 Proximate composition and energy content

Table 2 shows the proximate composition and energy content of maize, groundnut, spinach, and sweet potato.

**Table 2:** Proximate composition of experimental raw materials.

Compositions	Maize	Groundnut	Spinach	Orange-fleshed sweet potato
Moisture (%)	11.4 ± 0.2	4.6 ± 0.1	4.4 ± 0.06	3.1 ± 0.07
Fat (%)	10.48 ± 0.8	45.4 ± 0.8	4.63 ± 1.50	1.76 ± 0.03
Protein (%)	8.39 ± 0.86	28.3 ± 0.82	28.07 ± 0.10	8.97 ± 0.29
Ash (%)	1.21 ± 0.18	2.34 ± 0.02	14.63 ± 0.10	3.12 ± 0.1
Fiber (%)	0.62 ± 0.18	2.04 ± 0.07	6.84 ± 0.270	2.07 ± 0.1
Carbohydrate (%)	67.91 ± 0.80	17.3 ± 1.27	41.43 ± 1.7	80.98 ± 2.3
Energy (kcal. /100 g)	399.56 ± 5.64	591.02 ± 5.05	319.70 ± 6.80	375.60 ± 12.4

Values are mean ± SD, *n* = 3, and the results are expressed on a dry basis.

The moisture content of maize, groundnuts, spinach, and orange sweet potato measured 11.40, 4.62, 4.40, and 3.13%, respectively, while the fat content was 10.48, 45.4, 4.63, and 1.76%. Groundnuts have a higher fat content than the other raw materials, followed by maize. In terms of protein content, both groundnuts and spinach displayed the highest values at 28%, while maize and sweet potato had 8.3 and 8.9%, respectively. Spinach leaf flour has the highest ash content at 14.6%, followed by sweet potato at 3.1%. Additionally, the crude fiber content was recorded as 0.6, 2, 6.8, and 2% for maize, groundnuts, spinach, and orange-fleshed sweet potato, respectively. Similarly, the energy density indicates that groundnuts have the highest energy content at 514 kcal/100 g, while maize, spinach, and orange-fleshed sweet potato have 399, 395, and 304 kcal/100 g, respectively.

#### 3.1.2 Minerals, phytochemicals, and β-carotene

The calcium content of the raw materials was 5, 24.7, 1,080, and 230 mg/100 g for maize, groundnut, spinach, and sweet potato, respectively (Table 3). Spinach flour has a relatively higher calcium concentration compared to the others. Similarly, the iron content of spinach was the highest at 28.7 mg/100 g when compared to the values for maize (3.7 mg/100 g), groundnut (3.8 mg/100 g), and sweet potato (3.1 mg/100 g). In addition to calcium and iron, the concentrations of zinc (3.4 mg/100 g) and magnesium (394 mg/100 g) in spinach leaves were greater than those found in groundnuts.

**Table 3:** Minerals, phytochemicals, and β-carotene content of raw materials.

Compositions	Maize	Groundnut	Spinach	Orange-fleshed sweet potato
Ca (mg/100 g)	5.09 ± 0.28	24.7 ± 0.19	1,080 ± 33	230 ± 9.24
Fe (mg/100 g)	3.75 ± 0.34	3.88 ± 1.09	28.7 ± 1.47	3.19 ± 0.71
Zn (mg/100 g)	1.19 ± 0.11	2.29 ± 0.08	3.46 ± 0.13	0.85 ± 0.25
Mg (mg/100 g)	66.83 ± 3.92	145.1 ± 9.7	394.9 ± 0.9	105.9 ± 3.1
Tannin (μg/g)	0.77 ± 0.38	14.9 ± 1.2	39.09 ± 1.4	5.14 ± 0.3
Phytic (μg/g)	190.00 ± 0.01	241.00 ± 0.02	180.00 ± 0.02	110.00 ± 0.0
Total Phenol (μg/100 g)	3096.91 ± 30.83	4104.91 ± 19.01	3747.9 ± 212	2918.2 ± 241
β-carotene (μg/g)	3.6 ± 0.12	13.2 ± 1.1	331.6 ± 9.05	139.9 ± 26.1

Values are mean ± SD, *n* = 3, and the results are expressed on a dry basis.

Spinach has the highest level of tannins at 39 μg/g, compared to maize at 0.77 μg/g, groundnut at 14.9 μg/g, and sweet potato at 5.1 μg/g. Groundnut displayed the highest phenol content at 4104.91 μg/100 g, while spinach came in second at 3747 μg/100 g, followed by sweet potato at 2918 μg/100 g and maize at 2530 μg/100 g. In contrast, phytic acid content was measured at 190.00, 240.00, 180.00, and 110 μg/g for maize, groundnut, spinach leaves, and sweet potato, respectively. Similarly, β-carotene levels were 3.6, 13.2, 331.6, and 139.9 μg/g for maize, groundnut, spinach, and sweet potato, respectively.

#### 3.1.3 Functional properties of raw materials

The oil absorption capacities of maize, groundnut, spinach, and sweet potato flours are 2.15, 2.17, 2.3, and 2.02 mL/g, respectively. The results indicate that spinach flour has a marginally higher oil absorption capacity than the other flours (Table 4). The bulk density of the flours shows that sweet potato flour has a slightly higher value (0.66 g/mL) compared to maize (0.58), groundnut (0.48), and spinach (0.51 g/mL). Water absorption capacity values of 2.46, 2.32, 3.8, and 5.67 mL/g were recorded for maize, groundnut, spinach, and sweet potato, respectively, indicating that sweet potato flour has a greater water absorption capacity than the other raw materials. The result of the solubility index for maize, groundnut, spinach, and sweet potato were 4.3, 31.3, 21.8, and 16.9%, respectively.

**Table 4:** Functional properties of ingredients.

Compositions	Maize	Groundnut	Spinach	Orange-fleshed sweet potato
Oil absorption (mL/g)	2.15 ± 0.12	2.17 ± 0.03	2.32 ± 0.02	2.02 ± 0.0
Bulk density (g/mL)	0.58 ± 0.01	0.48 ± 0.0	0.51 ± 0.01	0.66 ± 0.02
Water absorption (mL/g)	2.46 ± 0.1	2.32 ± 0.29	3.8 ± 0.02	5.67 ± 0.22
Solubility Index (%)	4.37 ± 0.86	31.3 ± 2.43	21.8 ± 1.01	16.9 ± 1.18

Values are mean ± SD, *n* = 3, and the results are expressed on a dry basis.

### 3.2 Effect of processing methods on chemical composition of raw materials

#### 3.2.1 Effect of processing methods on proximate composition and energy content

**Figure 1:**
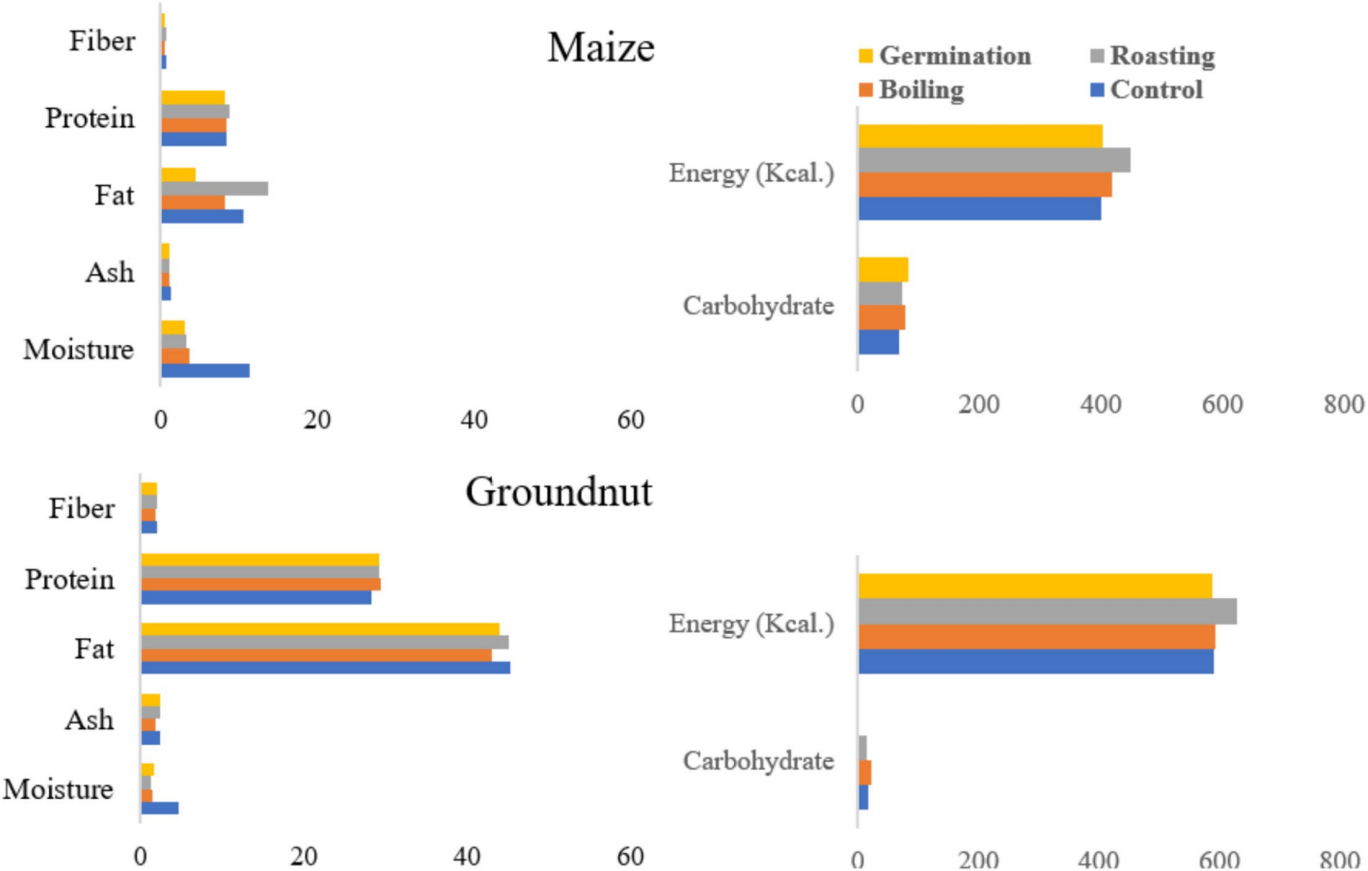
The effect of processing methods on the proximate composition and energy content of maize and groundnut flour.

Processing methods significantly influenced (*p ≤* 0.05) the moisture content of maize flour. The control showed a notably higher moisture content (11.4%) compared to the other samples, while germinated maize had the lowest moisture value (3.13%) (Figure 1). Although the moisture content was significantly affected by processing methods, no differences were observed between germinated and roasted maize flour. Similarly, moisture content varied significantly (*p* ≤ 0.05) among the processing methods for groundnut. The control’s moisture content was higher (4.61%) than that of the other samples obtained from boiling, roasting, and germination, which exhibited the lowest values of 1.5, 1.29, and 1.6%, respectively. Moisture is measured for various reasons, including legal requirements, economic significance, food quality, improved processing operations, and storage stability.

The crude fat content of maize was significantly affected by the processing methods (p ≤ 0.05). Roasted maize demonstrated the highest crude fat content at 13.7%, in comparison to all other processing methods, followed by a content of 10.48% (refer to Figure 1). Germination exhibited the lowest crude fat content, registering a value of 4.4%. Conversely, the processing methods did not exert a significant impact on the fat content of groundnut (*p* ≥ 0.05). The control sample recorded a fat content of 45.3%, followed by roasted samples at 45.16%, germinated samples at 43.9%, and boiled samples at 42.9%. Furthermore, processing methods have a considerable effect on the carbohydrate content of both maize and groundnut (*p* < 0.05). For maize, the maximum carbohydrate content attained was 82.24%, whereas for groundnut, it was measured at 23.08%.

#### 3.2.2 Effect of processing methods on physical and functional properties of raw material flour

Table 5 shows the effects of processing methods on the physical and functional qualities of maize and groundnut flour. The control and germinated maize flours exhibited higher oil absorption indices of 2.15 and 2.07 g/g, respectively. Similarly, the control groundnut flour showed a higher oil absorption index of 2.17. The water absorption indices for maize and groundnut flours ranged from 2.45 to 3.15 g/g and from 1.93 to 2.53 g/g, respectively. A higher water absorption index of 3.15 g/g was recorded for maize, while for groundnut, it stood at 2.53 g/g during boiling processing methods. Boiled maize flour displayed a significantly higher bulk density value of 0.62 g/mL, whereas the lowest value was noted for flours obtained from both roasting and germination. For groundnut flour, the maximum bulk density value of 0.61 g/mL was found in germinated flour. Germinated maize flour demonstrates a maximum solubility index of 8.31 compared to the other flours.

**Table 5:** Influence of processing methods on physical and functional properties of raw material flour.

Sample	Processing method	Functional property			
		Oil absorption (g/g)	Bulk density (g/mL)	Water Absorption Capacity (mL/g)	Solubility (%)
Maize	Control	2.15 ± 0.12^a^	0.58 ± 0.01^b^	2.46 ± 0.1^b^	4.37 ± 0.86^b^
	Boiling	1.99 ± 0.01^b^	0.62 ± 0.02^a^	3.15 ± 0.3^a^	3.16 ± 0.25^c^
	Roasting	1.93 ± 0.02^b^	0.5 ± 0.01^c^	2.49 ± 0.03^b^	4.29 ± 0.34^bc^
	Germination	2.07 ± 0.07^ab^	0.48 ± 0.01^c^	2.45 ± 0.01^b^	8.31 ± 0.33^a^
	CV (%)	4.12	3.2	6.573	12.31
	LSD	0.15	0.03	0.32	1.16
	Control	2.17 ± 0.03^a^	0.48 ± 0.0^c^	2.32 ± 0.29^a^	31.3 ± 2.43^a^
Groundnut	Boiling	1.82 ± 0.03^c^	0.54 ± 0.0^b^	2.53 ± 0.08^a^	22.9 ± 1.74^b^
	Roasting	1.82 ± 0.07^c^	0.5 ± 0.0.0b^c^	2.22 ± 0.05^ab^	30.7 ± 2.62^a^
	Germination	1.91 ± 0.19^b^	0.61 ± 0.05^a^	1.93 ± 0.05^b^	25 ± 1.77^b^
	CV (%)	0.24	6.04	8.35	9.69
	LSD	0.24	0.06	0.35	5.02

Values represent means ± standard deviation, *n* = 3, and the results are expressed on a dry basis. Means with the same superscript on the same column are not significantly.

Different (*p* > 0.05).

#### 3.2.3 Effect of processing methods on mineral content of maize and groundnut

**Figure 2:**
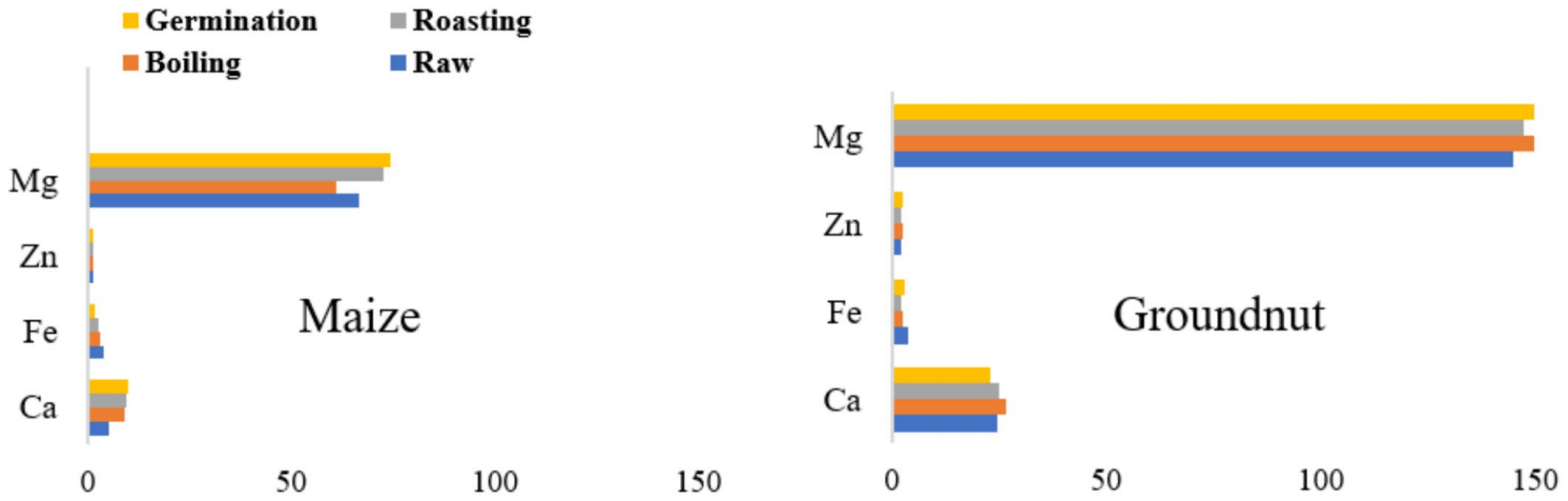
Effects of processing methods on the mineral content of maize and groundnut flour.

The mineral composition (mg/100 g) of maize and groundnut flour is shown in Figure 2. Raw maize flour (unprocessed) exhibited the lowest calcium content (5.09 mg/100 g), while the calcium content in maize flour showed no significant difference (*p* ≥ 0.05) across processing methods. The lowest value (1.8 mg/100 g) for iron was recorded in maize flour obtained from germination. Groundnut flour demonstrated the lowest calcium value (22.80 mg/100 g) and iron content (2.08 mg/100 g) from germinated and roasted flour, respectively. There were no significant changes (*p* ≥ 0.05) in mineral content across processing methods for groundnut flour.

#### 3.2.4 The impact of processing methods on the anti-nutritional factors and β-carotene content in maize and groundnuts

**Figure 3:**
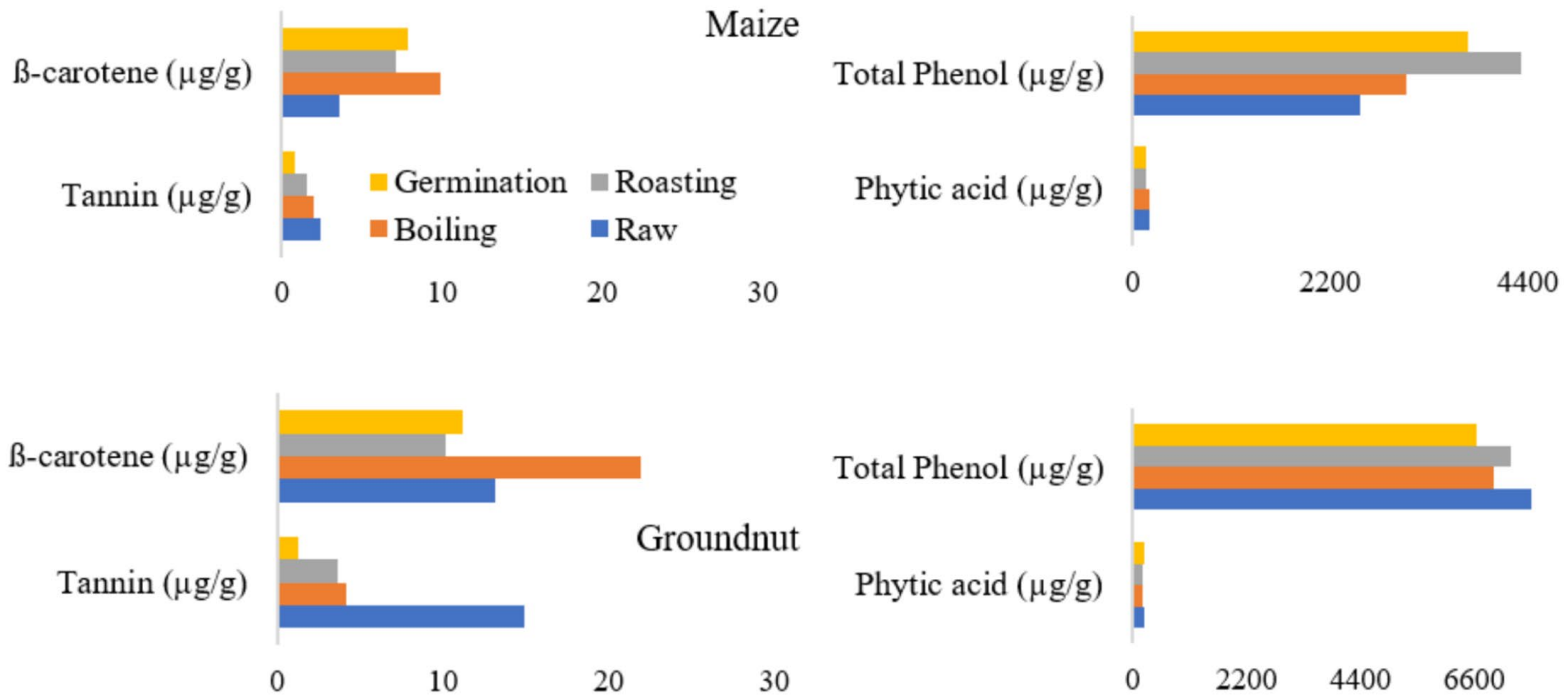
Effects of processing methods on anti-nutritional factors and β-carotene content in maize and groundnut flour.

The tannin content of maize and groundnut flours ranged from 0.77 to 2.46 μg/g and from 1.29 to 14.9 μg/g, respectively (Figure 3). The minimum value was recorded for both germinated maize and groundnut flour.

The phytic acid content of both maize and groundnut flour does not show a significant difference (*p* > 0.05) based on the processing methods. Similarly, the total phenol content of groundnut flour did not exhibit a significant difference (*p* ≤ 0.05) among the various processing methods. The β-carotene content in roasted maize (7.1 μg/g) and groundnut (10.2 μg/g) flour revealed a significant decrease.

### 3.3 Chemical composition of formulated infant food

#### 3.3.1 Proximate composition and energy content

The moisture content of the developed infant foods revealed no statistically significant difference (p ≥ 0.05) between the lowest moisture level of 5.14% (F8) and the maximum of 6.83% (F5) (Table 6). The ash content of the products varied from 2.56 to 3.64%, with significant differences observed between the products (*p* < 0.05). The highest ash value was recorded in formula F4 (3.64%), composed of germinated maize and groundnut flours. The highest ash content was found in a product formulated with maize, sweet potato, spinach, and groundnut in the ratios of 45, 25, 15, and 10%, respectively, while the lowest ash content (2.56%) came from a formulation with the ratios of 60, 10, 10, and 15%. The highest crude fiber values were 4.53, 5.00, and 4.45%, noted in formulas F3, F4, and F8, while the lowest values were 2.25, 2.56, and 3.01% in formulas F1, F2, and F5.

**Table 6:** Energy and proximate compositions of developed complementary food (100 g).

Formula (F)	Ash (%)	Moisture (%)	Fat (%)	Protein (%)	Fiber (%)	Carbohydrate (%)	Energy (Kcal)
F1	2.68 ± 0.16^d^	6.34 ± 1.21^a^	10.8 ± 0.65^a^	16.39 ± 0.71^a^	2.75 ± 0.20 ^c^	60.35 ± 0.81^c^	404.19 ± 2.67^ab^
F2	3.38 ± 0.03^b^	6.07 ± 2.58^a^	9.39 ± 0.34^a^	16.18 ± 1.31^a^	2.56 ± 0.07^c^	63.07 ± 2.06^ba^	401.60 ± 6.77^ab^
F3	2.89 ± 0.04^c^	4.95 ± 1.18^a^	10.5 ± 1.06^a^	15.68 ± 0.52^a^	4.53 ± 0.50^ab^	62.08 ± 2.21^a^	405.69 ± 3.3^ab^
F4	3.64 ± 0.06^a^	5.53 ± 0.28^a^	8.10 ± 0.39^a^	16.02 ± 0.04^a^	5.00 ± 0.63^a^	61.69 ± 0.87^c^	383.82 ± 2.77^b^
F5	2.56 ± 0.04^d^	6.83 ± 1.25^a^	12.4 ± 0.43^a^	15.60 ± 0.46^a^	3.01 ± 0.10^c^	60.12 ± 0.54^d^	412.56 ± 4.87^a^
F6	3.63 ± 0.06^a^	5.01 ± 1.63^a^	10.52 ± 0.42^a^	15.35 ± 0.38^a^	4.21 ± 0.44^b^	61.42 ± 0.49^d^	401.87 ± 4.55^ab^
F7	2.87 ± 0.12^c^	5.68 ± 1.4^a^	11.92 ± 0.60^a^	16.14 ± 0.83^a^	4.12 ± 0.11^b^	60.08 ± 1.38^bc^	412.21 ± 4.78^a^
F8	3.55 ± 0.10^a^	5.14 ± 1.0^a^	11.31 ± 0.24^a^	16.57 ± 0.52^a^	4.45 ± 0.46a^b^	59.61 ± 0.08^d^	406.64 ± 1.05^ab^
CV (%)	2.95	25.52	24.21	4.77	8.52	5.58	3.28
LSD	0.16	2.51	4.567	1.32	0.56	5.89	23.09

Values represent means ± standard deviation, *n* = 3, and the results are expressed on a dry basis. Means with the same superscript on the same column are not significantly.

Different (*p* > 0.05). F1 to F4 was developed from roasted maize and groundnut, sweet potato, and spinach leaf flour; F5 to F8 was developed from germinated maize and groundnut, sweet potato, and spinach leaf flour.

The crude protein content of the products ranged from 15.35 to 16.39%, indicating no statistical difference (*p* ≥ 0.05) among them. Both the carbohydrate and energy content of the developed complementary foods also showed no statistical difference (*p* ≥ 0.005) among the formulas, with the highest values being 63.07 for carbohydrates and 412.56 kcal for energy in F2 and F5, respectively. Similarly, the products exhibited a fat content ranging from 8.10 to 11.92%, with no statistical difference (*p* ≥ 0.005).

#### 3.3.2 Functional properties and anti-nutritional contents of formulated powder

The complementary food developed in this study yielded results ranging from 2.63 to 3.21 mL/g for the water absorption index (WAI). The lowest WAI values, 2.63 and 2.86 mL/g, were recorded at F1 and F2 (Table 7). The WAI measures the water-holding capacity of starch after it swells in excess water, corresponding to the weight of the gel formed and serving as an index of the degree of starch gelatinization. Additionally, the oil absorption index (OAI) for the developed complementary food ranged from 1.62 to 1.91 g/g.

**Table 7:** Functional properties of the prepared formula.

Code	OAI (g/g)	WAI (mL/g)	BD (g/mL)	SI (%)
F1	1.72 ± 0.07^bcd^	2.63 ± 0.03^c^	0.67 ± 0.01^c^	9.77 ± 0.96^e^
F2	1.62 ± 0.03^d^	2.86 ± 0.06^bc^	0.78 ± 0.01^a^	13.17 ± 1.59^cd^
F3	1.64 ± 0.06^d^	3.05 ± 0.18^ab^	0.74 ± 0.0^b^	14.4 ± 1.03^bc^
F4	1.68 ± 0.01^d^	3.07 ± 0.02^ab^	0.78 ± 0.01^a^	15.8 ± 0.11^ab^
F5	1.67 ± 0.06^d^	2.92 ± 0.29^b^	0.79 ± 0.02^a^	9.2 ± 0.85e
F6	1.91 ± 0.07^a^	2.96 ± 0.13^ab^	0.80 ± 0.01^a^	11.7 ± 1.22^d^
F7	1.81 ± 0.08^ab^	2.98 ± 0.04^ab^	0.80 ± 0.02^a^	15.3 ± 0.40^ab^
F8	1.8 ± 0.09^bc^	3.21 ± 0.18^a^	0.79 ± 0.01^a^	16.3 ± 0.56^a^
CV (%)	3.92	5.11	2.00	7.20
LSD	0.11	0.26	0.02	1.65

Values represent means ± standard deviation, *n* = 3, and the results are expressed on a dry basis. Means with the same superscript on the same column are not significantly different (*p* > 0.05). OAI, Oil absorption index. WAI, water absorption index BD, bulk density, and SI, solubility index. F1 to F4 was developed from roasted maize and groundnut, sweet potato, and spinach leaf flour; F5 to F8 was developed from germinated maize and groundnut, sweet potato, and spinach leaf flour.

The bulk density (BD) in this study ranged from 0.67 to 0.80 g/mL. The BD of germinated maize and groundnut composite products was lower than that of roasted maize. The solubility index (SI) in this study ranged from 9.2 to 16.30%. The highest WSI was recorded at 16.3, 15.80, and 15.30% for formulas F8, F4, and F7.

#### 3.3.3 Mineral content

The calcium content of the formulated foods ranged from 66.75 to 102.49 mg/100 g (Table 8). The highest calcium level was found in F6, which was made from maize, sweet potato, spinach, and groundnut in ratios of 55, 15, 15, and 10%, respectively.

**Table 8:** Mineral contents of the prepared formula (mg /100 g).

Code	Ca	Fe	Mg	Zn
F1	68.97 ± 0.73^c^	6.83 ± 0.52^a^	144.34 ± 0.58^de^	1.33 ± 0.17b^c^
F2	99.02 ± 0.83^ab^	8.33 ± 0.48^a^	181.73 ± 0.10^a^	1.47 ± 0.17^c^
F3	79.46 ± 1.28^abc^	6.64 ± 0.24^a^	155.09 ± 0.36^cde^	1.33 ± 0.23^c^
F4	90.98 ± 0.97^abc^	7.04 ± 0.53^a^	173.38 ± 0.25^ab^	1.50 ± 0.18^c^
F5	66.75 ± 0.619^c^	8.05 ± 0.62^a^	122.60 ± 0.14^e^	1.66 ± 0.86^c^
F6	102.48 ± 3.59^a^	8.50 ± 0.61^a^	143.92 ± 2.79^abc^	2.30 ± 0.50^ab^
F7	74.75 ± 0.70^abc^	8.03 ± 0.33^a^	143.9 ± 2.24^de^	2.48 ± 0.04^a^
F8	84.63 ± 0.96^ab^	10.36 ± 1.03^ab^	160.44 ± 2.24^bcd^	1.99 ± 0.21^abc^
CV (%)	17.67	11.73	6.47	21.96
LSD	25.76	2.94	17.50	0.67

Values represent means ± standard deviation, *n* = 3, and the results are expressed on a dry basis. Means with the same superscript on the same column are not significantly different (*p* > 0.05). F1 to F4 was developed from roasted maize and groundnut, sweet potato, and spinach leaf flour; F5 to F8 was developed from germinated maize and groundnut, sweet potato, and spinach leaf flour.

The iron content of the developed formulas showed no variation (*p* > 0.05) among the products, with the highest and lowest iron levels being 10.36 mg/100 g for F8 and 6.8 mg/100 g for F1, respectively. The highest zinc value, at 2.48 mg/100 g, was found in F7, while F1 and F3 each had the lowest zinc content of 1.33 mg/100 g. The magnesium content of the formulated food ranged from 122.60 mg/100 g (F5) to 181 mg/100 g (F2).

#### 3.3.4 β-carotene content and anti-nutritional factors of the formula

In this study, the β-carotene content of the developed food ranged from 113.40 to 197.53 μg/g. The highest β-carotene content, 197.53 μg/g, was obtained from F8 (Table 9). The proportions of maize, sweet potato, spinach, and groundnut that showed the highest β-carotene content were in the ratios of 55, 25, 15, and 10%, respectively.

**Table 9:** Anti-nutritional and β-carotene content of the prepared formula.

Code	Tannin (μg/g)	Phytic (μg/g)	Phenol (μg/100 g)	β-carotene (μg/g)
F1	2.48 ± 0.17^c^	190.00 ± 0.01^ab^	30996.60 ± 32.38^e^	120.32 ± 0.78^cd^
F2	5.79 ± 1.27^a^	160.00 ± 0.01^de^	3530.46 ± 90.07^de^	172.57 ± 6.83^b^
F3	4.50 ± 0.37^a^	170.00 ± 0.01^bc^	4089.63 ± 102.74^cd^	126.99 ± 5.89^c^
F4	4.62 ± 0.79^a^	150.00 ± 0.01^e^	4473.47 ± 84.83^c^	162.33 ± 0.03^ab^
F5	2.22 ± 0.20^c^	170.00 ± 0.0^abc^	5173.60 ± 318.89^ab^	113.4 ± 16.9^d^
F6	5.16 ± 0.51^a^	190.00 ± 0.01^a^	4618.00 ± 280.71^bc^	186.07 ± 5.47^ab^
F7	2.98 ± 0.73^bc^	170.00 ± 0.0^cd^	5580.11 ± 184.03^a^	131.98 ± 3.54^c^
F8	4.40 ± 1.49^ab^	160.00 ± 0.01^de^	5664.10 ± 87.34^a^	197.53 ± 4.34^a^
CV (%)	20.64	4.168	7.55	5.06
LSD	1.43	0.01	592.31	13.52

Values represent means ± standard deviation, *n* = 3, and the results are expressed on a dry basis. Means with the same superscript on the same column are not significantly different (*p* > 0.05). F1 to F4 was developed from roasted maize and groundnut, sweet potato, and spinach leaf flour; F5 to F8 was developed from germinated maize and groundnut, sweet potato, and spinach leaf flour.

The total phenol content of the product ranged from 30996.60 (F1) to 5664.10 mg/100 g (F8), indicating significant variation among products. All developed complementary foods showed considerable differences in condensed tannin contents, ranging from 2.22 to 5.79 mg/g. The highest amount of condensed tannin was found in F2, while the lowest was observed in F5. Similarly, the phytate content of the products was tested and ranged from 150 to 190.00 μg/100 g, with the highest values attributed to F4 and F6, respectively.

#### 3.3.5 Sensory acceptability

The sensory acceptability of the products exhibited significant differences (*p* ≤ 0.05) among the developed products. The color scores varied significantly (*p* ≤ 0.05) among the infant formulas, with high acceptability for F1, F5, and F3 with scores of 8.43, 8.48, and 8.43, respectively. The last color score was recorded for F6 with a score of 7.42 (slightly liked) (Table 10).

**Table 10:** Sensory acceptability score of different formulas.

Formula	Color	Taste	Aroma	Texture	Overall acceptability
F1	8.43 ± 0.6^a^	8.91 ± 0.1^a^	8.91 ± 0.1^a^	8.74 ± 0.5^a^	8.91 ± 0.3^a^
F2	7.58 ± 0.6^d^	8.22 ± 0.6^c^	8.05 ± 0.4^b^	8.16 ± 0.8^b^	8.42 ± 0.7^bc^
F3	8.43 ± 0.8^a^	8.20 ± 0.6^bc^	7.69 ± 0.5^b^	8.24 ± 0.7^cd^	7.90 ± 0.5^c^
F4	7.88 ± 0.6^bc^	8.32 ± 0.6^b^	8.21 ± 0.6^b^	8.12 ± 0.7^bc^	8.23 ± 0.6^c^
F5	8.48 ± 0.8^a^	8.98 ± 0.1^a^	8.90 ± 0.1^a^	8.98 ± 0.1^a^	8.90 ± 0.3^a^
F6	7.42 ± 0.7^d^	8.10 ± 0.6^c^	8.32 ± 0.3^b^	7.96 ± 0.8^b^	8.28 ± 0.7^b^
F7	7.60 ± 0.7^cd^	8.12 ± 0.8^d^	7.76 ± 0.6^b^	7.50 ± 1.1^d^	7.70 ± 0.8^c^
F8	7.98 ± 0.5^b^	8.06 ± 0.7^c^	7.74 ± 0.8^b^	8.04 ± 0.6^bc^	8.02 ± 0.5^c^
LCD	0.56	0.23	0.54	0.58	0.46
CV	17.40	16.50	12.20	14.0	11.50

Values represent means ± standard deviation, *n* = 30, and the results are expressed on a dry basis. Means with the same superscript on the same column are not significantly different (*p* > 0.05). F1 to F4 was developed from roasted maize and groundnut, sweet potato, and spinach leaf flour; F5 to F8 was developed from germinated maize and groundnut, sweet potato, and spinach leaf flour.

The taste of the products significantly varied (*p* ≤ 0.05) among the different formulas. The infant formulas that received the highest taste ratings of ‘like extremely’ were F1 and F5, with scores of 8.91 and 8.98, respectively. Additionally, the other infant formula products were ‘strongly liked’ by the panelists. The lowest taste score, 8.06, was recorded for F8.

Similarly, aroma, similar to color and taste, was observed to vary significantly (*p* ≤ 0.05) among the developed complementary foods. The highest score of aromas was recorded for F1 (8.91) and F5 (8.98), with panelists rating them as ‘like extremely,’ while the lowest score was for F3 (7.69), F7 (7.76), and F8 (7.74). Furthermore, sensory acceptability results indicated that texture also varied significantly (*p* ≤ 0.05) among the products. F1 and F5 received the highest acceptability scores, at 8.74 and 8.98, respectively, whereas F6 and F7 had the lowest scores.

The overall acceptability of the infant formulas showed statistical variability (*p* ≤ 0.05). F1 and F5 received the highest overall acceptability scores, rated as ‘extremely liked,’ whereas F3 and F7 had the lowest score compared to the remaining infant formulas.

## 4 Discussion

All raw materials, except for maize with 11%, showed moisture content below 5% (Table 2). Nyam et al. (32) noted that low moisture content is one of the most significant factors affecting a product’s shelf life. Foods with moisture levels of 14% or higher are more vulnerable to microbial attack and fungal growth, while lower levels inhibit microbial growth and extend shelf life (33). It has been reported that the oil content of maize can reach up to 5%, but in the current results, it was found to be 10.4%. This variation may arise from different varieties and growing conditions (34). According to the literature, the minimum carbohydrate content of food is 35% (35), implying that, except for groundnuts, all the raw materials provide adequate carbohydrates.

Micronutrients such as iron, magnesium, zinc, and calcium are crucial for ensuring optimal health and growth. Foods that are rich in calcium are essential for the health and development of infants (36). Iron plays a significant role in the composition of blood and in enzymes that are responsible for electron transfer (79). Therefore, the raw materials used in this study may provide potential health benefits. The slight variation in mineral content compared to existing literature can be attributed to agricultural practices, cultivars, ecology, and soil chemistry, all of which influence the mineral content of crops (37).

Tannins, however, can be considered anti-nutrients that bind to essential minerals such as iron, significantly reducing their availability. Therefore, they should be minimized in most foods meant for mineral supplementation (38). It is clear that children, especially preschoolers, require a high intake of nutrients, and vitamin A is a micronutrient that deserves special attention (39).

The oil absorption capacity of food is due to the physical entrapment of oil. This property is crucial for flavor retention and the mouthfeel of foods (40). Bulk density measures the heaviness of solid samples, which is essential for determining packaging needs, material handling, and applications within the food industry (41). Water absorption capacity refers to flour’s ability to absorb water and swell, enhancing food consistency (42). It serves as a useful indication of whether flour or isolates can be incorporated into aqueous food formulations (43). The solubility of flour rises in direct proportion to its swelling; high solubility frequently leads to notable leaching (44).

Processing methods reduce the moisture content of raw materials, which can limit microbial growth and is thus an important factor in food preservation (45). Such processes, including boiling and germination, have been shown to significantly reduce fat content. During germination, metabolic activities are enhanced, leading to the conversion of molecules, including fat, into metabolites through the action of lipase (46–49). Boiling lowers fat content by leaching fat molecules into the boiling water (50).

The decrease in oil absorption index values for both maize and groundnut flour may be attributed to the likely occurrence of irreversible denaturation caused by heating. Heating could have destroyed protein, leading to a reduction in both OAI and WAI (51). The high WAI of the flour indicates that it can be used in the formulation of various foods, such as dough and bakery products (52).

The increase in the WAI has always been associated with an increase in amylose leaching and solubility, as well as the degradation of the starch crystalline structure. Flour with a high WAI may contain more hydrophilic components, such as polysaccharides. The high bulk density of flour indicates its suitability for food preparations. Conversely, low bulk density can be beneficial in the formulation of complementary foods (53). Therefore, in this study, the lowest bulk density of maize and groundnut flour obtained from roasting suggests its suitability for infant food formulation (54).

The high solubility index of germinated maize flour may be due to hydrolyzed starch and increased sugar levels that develop during germination. Furthermore, protein solubility could be affected by heat treatment (52).

Boiling reduces the calcium content compared to roasting and germination, as boiling water can leach the minerals (55). The high levels and availability of these minerals in groundnuts indicate that groundnut consumption supports bone formation, blood clotting, muscle contraction, and certain enzymes involved in metabolic processes (56).

Figure 3 shows an increase in beta-carotene and phenol content in maize following processing techniques such as boiling, roasting, and germination, compared to their raw forms. Notably, levels of tannins and phytic acid show a reduction after processing relative to the raw versions. The observed increase in beta-carotene can be attributed to the disruption of plant cell walls caused by these processing methods. The decrease in tannin content may result from the leaching of polyphenols into the soaking water and the effects of thermal treatment (57). A lower tannin content in food is generally considered beneficial, as it affects protein digestibility and can reduce essential amino acids by forming tannin-protein complexes, thereby diminishing the bioavailability of proteins (58). Moreover, germination is regarded as a very effective technique for reducing the antinutrient components found in plant-based foods. Phytic acid, a secondary compound, naturally accumulates in plant seeds, especially in legumes, peanuts, cereals, and oilseeds, and is typically present in all plant-based food sources (59).

The fiber content is nearly equivalent to the recommendations of the Ethiopian Standard Agency for infant complementary foods (80). The daily recommended allowance of crude fiber in complementary food should exceed 5%. Therefore, all the complementary foods processed in this study satisfy this requirement.

All developed infant foods were found to have improved and met the protein level requirements of 14.00 and 15.00 g/100 g according to Ethiopian standard agency guidelines for formulated complementary foods. The Codex Alimentarius Commission’s requirements for supplemental foods are 5.2 g/day for 6–8 months, 6.7 g/day for 9–11 months, and 9.1 g/day for 12–16 months, assuming adequate intake of breast milk for babies aged 23 months. Protein requirements for newborns are 9.1 g/day for 6–8 months, 9.6 g/day for 9–11 months, and 10.9 g/day for 12–23 months, assuming insufficient intake of breast milk (60). A balanced energy intake is recommended for proper development and body function. In many commercial complementary foods, the energy density typically ranges from 0.6 to 1.0 kcal/g, while the energy requirement for infants is approximately 615 kcal/day. Therefore, the currently developed formula provides 50% of the daily energy requirement for infants aged 6 months and older (61). The crude fat content of the developed complementary formula falls within the range of 8.10 to 12.70% (62).

WAI relies on the availability of hydrophilic groups that bind water molecules and on the gel-forming capability of macromolecules (63). Water absorption capacity is related to the composition and physical properties of starch granules and fiber, as they both exhibit water absorption capacity (17). The oil absorption index is essential because oil acts as a flavor retainer and enhances the mouthfeel of foods, thereby improving palatability (30, 64). Bulk density is a functional characteristic of foods, including flours, powders, and granules (or food ingredients) (63, 65). The lower bulk density observed in this study may result from germination, which softens the seeds, facilitates milling, and leads to smaller particle sizes compared to boiling (66). The solubility index reflects the solubility of biomolecules (such as starches, water-soluble fibers, proteins, and sugars) in excess water. A lower solubility indicates minimal degradation of starch, resulting in fewer soluble molecules in food (10, 67).

Calcium-rich foods are vital for the health and development of infants (36, 68, 69). The recommended daily intake of calcium for babies ranges from 210 to 500 mg, and the calcium concentration in commercial formulas is 80 mg per 100 g (60). The currently developed infant food contains as much as 99.02 mg of calcium per 100 g. For infants aged 6–8 months, the recommended daily intake of iron is 0.27 mg; for those aged 9–11 months, it is 11 mg; and for infants aged 12–23 months, it is 7 mg (70). The zinc content of the formulated complementary food varies from 1.33 to 2.48 mg per 100 g, which is consistent with the range reported by Ijarotimi et al. (71). The recommended daily intake of zinc for infants aged 6–8 months is 2 mg. For 9–11 months, it is 3 mg, and for 12–23 months, it is also 3 mg (60). All formulas contain higher magnesium levels compared to the daily recommended amount of 40 to 60 mg per 100 g (72).

High levels of beta carotene (5,300 μg/100 g) have been reported in spinach leaves (73), and incorporating spinach as an ingredient in infant food formulation plays a significant role. The recommended dietary allowance (RDA) of β-carotene is 400 mcg RAE for infants from birth to 6 months, 500 mcg RAE for 7–12 months, and 300 mcg RAE for children aged 1–3 years (74). The increased phenol content in spinach powder contributes to a higher concentration in the developed infant formula. However, from a nutritional standpoint, the main concern with phenols in dietary proteins is their potential to reduce digestibility and nutritional value (75, 76). The majority of developed formulas contain lower condensed tannin levels compared to the recommended intake (560 mg/100 g), with the exception of F2, which showed 5.79 mg/g (76). Phytic acid serves as an antinutrient that decreases the bioavailability of minerals and impairs protein digestibility (77). The phytate concentration in the currently developed food is low in comparison to the recommended daily intake (300–500 mg/day) (60). The antinutrients present in the formulated complementary foods are lower than those found in the raw materials used for formulation and are significantly less than those reported by Ohizua et al. (78).

All the formulated products received sensory acceptability scores ranging from ‘moderately liked’ to ‘extremely liked’ across all sensory attributes. While all formulations were accepted, F1 and F5 stood out with the highest overall acceptability, achieving a score of ‘extremely liked.’

## 5 Strengths and limitations

The output of the current project creates opportunities for producing infant formula from locally available, nutrient-rich food sources. Additionally, it may indicate the potential for engaging in business by manufacturing commercial formulas for the local community. Processing conditions such as boiling, germinating, and roasting, along with blending ratios, significantly affect the nutritional value and sensory acceptance of the prepared food. However, the project faces limitations in analyzing the amino acid profile, investigating protein digestibility, conducting shelf-life studies, and assessing the microbial load of the developed formula.

## 6 Conclusion

Among the complementary foods developed in this research, one formula contained 60, 15, 10, and 10% roasted maize, roasted groundnut, orange-fleshed sweet potato flour, and spinach flour, respectively. These infant formulas displayed a commendable chemical composition (including proximate composition, minerals, and reduced anti-nutritional factors) and overall sensory acceptability. Overall, the study demonstrated the potential to produce nutritionally enriched infant food from locally available and affordable food sources using simple processing technology. Therefore, profit-oriented individuals can engage in production for broader distribution.

## Data Availability

The raw data supporting the conclusions of this article will be made available by the authors without undue reservation.
